# Progress on Incorporating Zeolites in Matrimid^®^5218 Mixed Matrix Membranes towards Gas Separation

**DOI:** 10.3390/membranes8020030

**Published:** 2018-06-14

**Authors:** Roberto Castro-Muñoz, Vlastimil Fíla

**Affiliations:** Department of Inorganic Technology, University of Chemistry and Technology Prague, Technická 5, 166 28 Prague 6, Czech Republic; vlastimil.fila@vscht.cz

**Keywords:** Matrimid^®^5218, zeolites, gas separation, mixed matrix membranes (MMMs)

## Abstract

Membranes, as perm-selective barriers, have been widely applied for gas separation applications. Since some time ago, pure polymers have been used mainly for the preparation of membranes, considering different kinds of polymers for such preparation. At this point, polyimides (e.g., Matrimid^®^5218) are probably one of the most considered polymers for this purpose. However, the limitation on the performance relationship of polymeric membranes has promoted their enhancement through the incorporation of different inorganic materials (e.g., zeolites) into their matrix. Therefore, the aim of this work is to provide an overview about the progress of zeolite embedding in Matrimid^®^5218, aiming at the preparation of mixed matrix membranes for gas separation. Particular attention is paid to the relevant experimental results and current findings. Finally, we describe the prospects and future trends in the field.

## 1. Introduction

**Figure 1:**
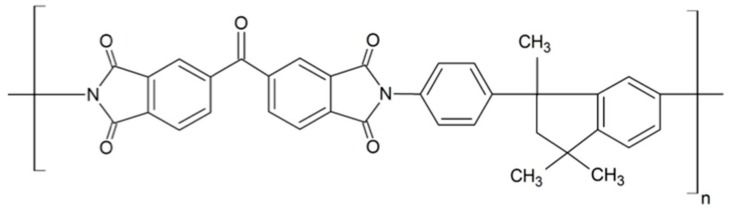
Chemical structure of the repeating unit of Matrimid^®^5218.

Matrimid^®^5218 is a commercially available thermoplastic polyimide (PI), which is obtained by polycondensation of 3,3′,4,4′-benzophenone tetracarboxylic dianhydride (BTDA) and 5,6-amino-1-(4′-aminophenyl)-1,3,3-trimethylindane (DAPI), producing 3,3′-4,4′-benzophenone tetracarboxylic-dianhydride diaminophenylindane (BTDA-DAPI) [1,2]. It is soluble in a variety of common solvents (i.e., CH_2_Cl_2_, CHCl_3_, THF, dimethylacetamide, dimethylformamide, n-methyl-2-pyrrolidone). The chemical structure of Matrimid^®^5218 can be widely found elsewhere (see Figure 1).

Generally, the use of this PI allows us to prepare membranes that possess excellent physical and chemical resistance as well as excellent thermal stability (T_g_ = 305–315 °C). Matrimid^®^-based membranes, together with other commercial polymers, have been applied for permeating different gases (i.e., CO_2_, H_2_, CH_4_, C_2_H_4_, C_2_H_6_) [3,4]. All these gas testings, either singly or in gas mixtures, aim to evaluate the performances of membranes towards several separations, such as H_2_/N_2_, H_2_/CO, H_2_/CO_2_, H_2_/hydrocarbons, N_2_/O_2_, CO_2_/air, CO_2_/CH_4_, and CO_2_/H_2_. Such applications have a clear point related to facilitating specific chemical processes, e.g., oxygen enrichment of air, hydrogen recovery, natural gas separation and removal of volatile components from gas effluent streams, and CO_2_ capture from flue gas, biogas, and syngas [5,6].

**Figure 2:**
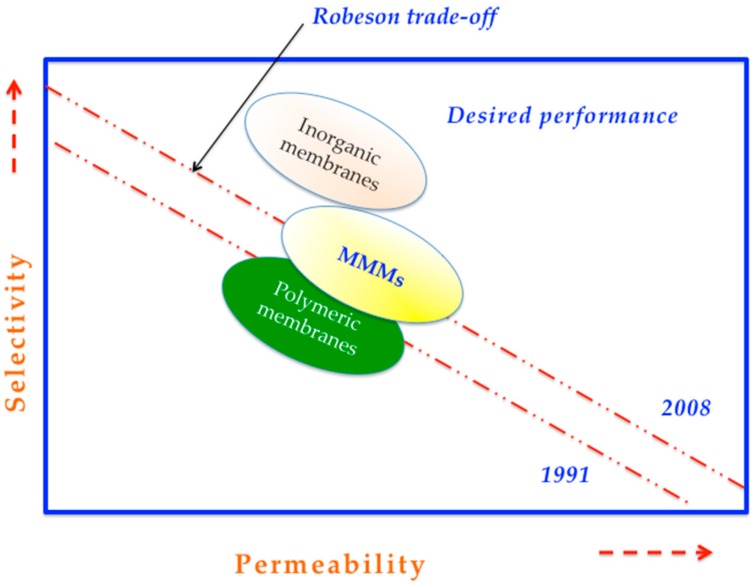
General drawing of Robeson relationship of polymeric membranes, inorganic and mixed matrix membranes (MMMs), and the desired performance [11,12,13].

Actually, this PI has been widely used as polymer matrix for preparing membranes due to the fact that its chains are imperfectly packed, creating an excess of free volume by means of microscopic voids [7], but its free volume is still considered relatively low (about 0.17). This characteristic has led it to efficiently perform the separation of some gas pairs. However, as is well known, highly selective polymers do not demonstrate high permeability performances, and highly permeable polymers are not selective enough. This particular limitation does not allow us to overcome the Robeson relationship, which was first proposed in 1991 [8] and revised in 2008 [9]. This describes the performances of polymeric membranes in terms of selectivity and permeability, as Figure 2 depicts. Moreover, the description also shows the desired performance that is sought by researchers. Matrimid^®^ membranes have been satisfactorily demonstrated to be close to overcoming the Robeson trade-off (1991) toward specific separations, i.e., CO_2_/CH_4_ and O_2_/N_2_ [10].

One of the current approaches, trying to reach this desired region, is the incorporation of inorganic materials into the Matrimid^®^ matrix. To date, different types of materials, such as metal–organic frameworks (MOFs) (e.g., ZIF-8, MIL-53, [Cu_3_(BTC)_2_]), silicas, carbon nanotubes (CNTs), graphene oxide, and zeolites, have been incorporated [4]. Table 1 provides the main features of some of these filling materials that have been proposed for the preparation of MMMs. Concerning zeolites, many reports have reported the favorable effects of using zeolites and zeolite-based materials as the dispersed phase to enhance the permeability and selectivity of several polymers for gas separation of different gas pairs.

Particularly, the aim here is to provide a critical overview of the literature inputs on incorporating zeolites into Matrimid^®^ for gas separation. Furthermore, a clear outlook is given on the progress of developments, as well as the prospects and future trends of the use of Matrimid^®^ for gas separation applications. Finally, fundamentals about zeolites in membrane separation applications are also given.

## 2. Fundamentals of Zeolites

**Figure 3:**
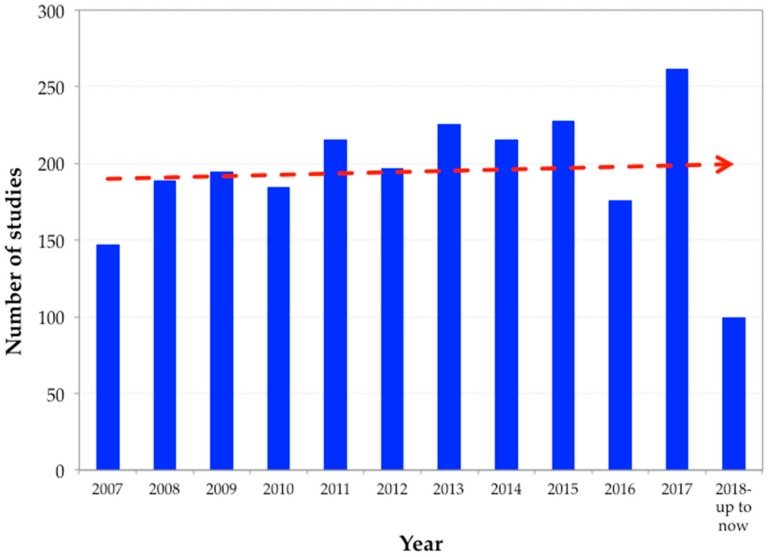
Evolution of the number of studies over the last decade using zeolites for separation applications (source: wwww.scopus.com, 8 May 2018).

Zeolites and zeolite-based materials are progressively finding new and different applications. Among all these applications, the use of zeolites for separating components can be surely found, e.g., being part of selective barrier-like membranes. For example, Figure 3 shows the evolution of the number of studies published over the last decade with the use of zeolite incorporation in membranes for different membrane separation applications. It can be seen that they have been continuously taken into account; indeed, researchers’ attention to them for such purposes is increasing. To date, zeolites have been considered for different membrane separation applications such as gas separation [15], pervaporation [16,17,18], microfiltration [19], ultrafiltration [20], nanofiltration [21], desalination [22], water treatment [23,24], and membrane distillation [25]. Either natural or artificial, zeolites are used for these applications thanks to their unique ion-exchange [26], adsorption, catalytic, and molecular sieving features. Moreover, zeolites are also commercially used as absorbents [27], detergents [28], and catalysts [29], to mention just a few. Currently, zeolites and zeolite-based materials continue to find more and new uses in commercial applications [30,31].

**Figure 4:**
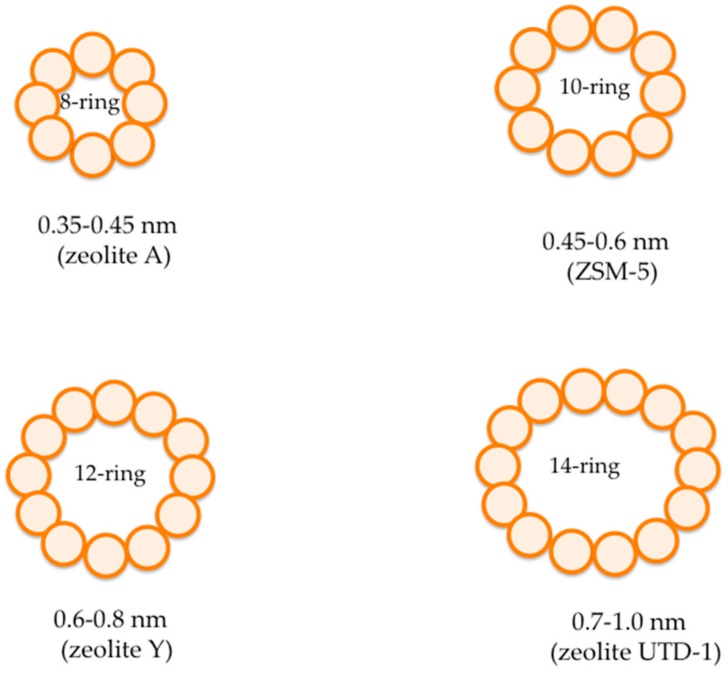
General drawing of typical zeolite pore sizes with oxygen packing models. Adapted from Flanigen et al. [32].

The interest in these materials can be related to their solid’s uniformity; this means that their use in macroscale can be reached according to their properties, which are controlled by their material chemistry at the atomic–molecular scale [31]. Zeolites are crystalline aluminosilicate materials, which can be formed by elements including potassium, sodium, magnesium, and calcium [32]. The general chemical formula of zeolites is represented by

$$M_{2/n}●{\mathrm{Al}}_{2}O_{3}●y{\mathrm{SiO}}_{2}{}_{}●wH_{2}O$$

where *n* is the valence of cation *M*, *w* is the number of water molecules, and *y* is ranged from 2 to 10. Basically, zeolites display a complex structure having a crystalline inorganic three-dimensional structure, and a four-connected framework of AlO_4_ and SiO_4_ tetrahedra linked to each other by sharing of an oxygen ion [33]. Thanks to the presence of AlO_4_ tetrahedra, the frameworks tend to have a negative charge, which is compensated for by alkali (e.g., Na, K) or earth-alkali (e.g., Mg, Ca) cations placed in the micropores [33]. The framework structure presents channels that are filled by the cations and water molecules. According to Flanigen et al. [32], there are over 70 novel different frameworks known, while more than 150 zeolites have been chemically synthesized, such as zeolites X, Y, A, and ZSM-5. The pore sizes of zeolites are between 0.3 and 1 nm, with pore volumes of about 0.10–0.35 cc/g. For instance, Figure 4 shows a typical zeolite pore size using oxygen packing models.

Particularly, some zeolites have small pore sizes with 8-ring pores of free diameters between 0.30–0.45 nm, and zeolite A possesses pores formed by 10-ring pores (free diameter of 0.45–0.60 nm). On the contrary, ZSM-5 possesses large pores with 12-ring pores (free diameter of 0.60–0.80 nm) [32].

Based on all these structural features, zeolites have been considered as a filling material for polymeric membranes to enhance their separation performance in any membrane separation application. When dealing with membrane gas separation, zeolites have been incorporated in several polymers such as polyether block amide [34], polysulfone (PSF) [35], poly(ether ether ketone) [36], polyurethane [37], and polyimides (PI) [38]. In particular, Matrimid^®^5218 has been one of the most sought-after PI to carry out the separation of gases. The properties of this commercial PI in prepared mixed matrix membranes are addressed in the following section.

## 3. The Concept of Mixed Matrix Membranes (MMMs) Incorporating Zeolites

Table 2 shows the main features of Matrimid^®^ membranes in comparison with those of other polymers commonly used for gas separation.

**Figure 5:**
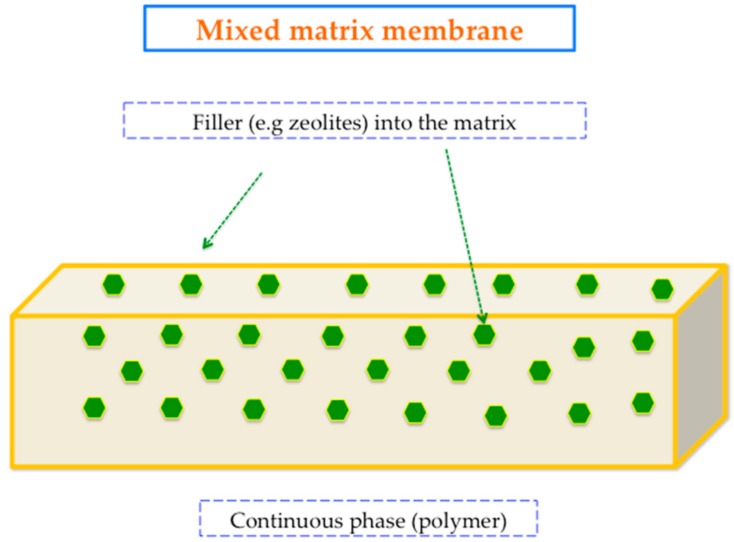
Mixed matrix membrane representation.

It can be seen that this PI tends to have low permeability values but acceptable selectivities for several gas pairs, which are totally associated with its low free volume. Basically, the low permeability is the key point that has encouraged the approach of preparation of mixed matrix membranes (MMMs) based on Matrimid^®^. The MMMs are conceptualized as the dispersion of organic–inorganic particles (so-called “fillers”) in a continuous polymeric matrix. Figure 5 displays a general scheme of a typical MMM.

The advantage of the MMMs is that they combine the ease of polymer film manufacture with the high selectivity and permeability of inorganic materials. The point of including inorganic materials is due to the fact that they have been demonstrated to positively shift the performance location of pristine membranes on the Robeson trade-off (see Figure 2); for example, inorganic membranes display high selectivity and thermal stability. In fact, the incorporation of these materials into polymeric membranes could also provide high temperature and pressure resistance to the final MMMs. For instance, the thermal decomposition of MMMs (using zeolite NaY-Matrimid^®^) has been reported to be higher than those of pure Matrimid^®^ membranes [39]. This is because a strong interaction of polymer–filler, formed by hydrogen bonds, is obtained, limiting the thermal motion of polymer; by this, the needed energy for polymer chain movement–segmentation is increased. Zeolites have been the first fillers to be proposed in MMMs for gas separation [40]; this is because they are a type of materials with a uniform pore system with molecule-sized dimensions, high porosity, large surface areas, excellent thermal and chemical stability [5,41], and easy regenerability [33]. They are particularly promising materials for the preparation of molecular-sieving membranes, being capable of separating gases in industrial conditions. Specific zeolites, such as molecular-sieve deca-dodecasil 3 rhombohedral (DDR)-type zeolite [42] and SSZ-13 [43], have shown hydrothermal stability at such conditions. Moreover, narrow-pore zeolites are able to separate molecules with relatively similar kinetic diameters, e.g., O_2_ and N_2_ [44]. In particular, the permeation in any zeolite starts with the adsorption of the gas molecules onto the zeolite pore surface. Certainly, adsorption affinity is crucial to the overall separation performance.

Depending on the type and polarity of the zeolites, they tend to exhibit adsorption selectivity; this means that the more strongly adsorbing component of a mixture disturbs or blocks permeation of other components for which zeolite channels remain not easily accessible. This makes adsorption-based separations particularly effective when a strong adsorbate has to be removed, e.g., CO_2_ capture. Fundamentally, the adsorption of certain gas molecules on the surface of a given zeolite material depends on the adsorbate and the adsorbent. At this point, the adsorbate parameters have to be strongly taken into account, e.g., polarizability and the dipole/quadrupole moments [5]. For instance, Table 3 displays some of these parameters for some gas molecules.

Moreover, the permeation of gases through zeolites is also governed by surface diffusion; this means that molecules hop from one adsorption site to another. Generally, the flux across zeolites combines surface and gas diffusion, while the separation mechanisms in zeolites can be described as follows: (i) *Adsorption selectivity* appears when the adsorption of one component is stronger than that of the others, and generally takes place a low temperature; (ii) *Diffusion selectivity* appears when the molecules of a specific component are smaller and, thus, their diffusivity in zeolite pores (commonly micropores) is faster than the others. This is practically promoted by increasing the temperature; (iii) *Molecular sieving* generally concerns the size exclusion when one component can clearly minimally or not at all penetrate through zeolite micropores [5,45]. When dealing with the type of zeolite, it is well known that the silicon/aluminum (Si/Al) ratio determines its properties, e.g., hydrophilicity, and particle size [46].

Considering all these separation features of zeolite, the crucial point of incorporating zeolites into Matrimid^®^ is mainly to promote the enhancement of its gas separation performance, which has been already reported. This is because zeolites (e.g., NaY) in Matrimid^®^ lead to a shift in the interstitial chain–chain distance and free volume distribution toward a looser and broader structure [39], producing an enhanced separation performance. The following section provides the recent developments reporting the effect of adding zeolites into this commercial PI.

## 4. Gas Transport Mechanism in MMMs

Typically, the mass transport through a membrane is described by Equation (1), as follows:

(1) $$J_{i}=-L_{i}\frac{d(\mu_{i})}{d(x)}$$

where *L_i_* is known as the coefficient of proportionality which links the chemical potential driving force to flux, and *dµ_i_*/*dx* is the chemical potential gradient of component *i* [47]. Dense polymeric membranes are used in gas separation processes, where the well-known solution–diffusion model describes the permeation across such membranes. Taking into account assumptions of the solution–diffusion mechanism, the simplification of Equation (1) in the form of Fick’s law (Equation (2)) [48], where the flux is related to the gradient of concentration, is generally accepted [49]. The flux (*J*) of component *i* is described by

(2) $$J_{i}=-D_{i}\frac{d(C_{i})}{d(x)}$$

where *dc_i_*/*dx* is the concentration gradient of component *i*, while *D_i_* is the diffusion coefficient (m^2^/s) which expresses the transport of individual molecules. When dealing with the use of porous membranes, e.g., zeolitic or inorganic membranes, the gas molecule diffuses from the high-pressure to the low-pressure side. Certainly, the various mass transfer mechanisms are contributing to the overall mass transfer of gas; however, in the case of a porous membrane, the description is more complex. For instance, if the pores are 0.1 µm or larger, gas permeation takes place by convective flow described by Poiseuille’s law and no separation is obtained between various gaseous components. However, if the pores are smaller and/or when the gas pressure is reduced, the mean free path of diffusing molecules becomes comparable or larger than the pore size. Diffusing gas molecules have more collisions with the wall of the pore than with other gas molecules (so-called Knudsen diffusion model). The selectivity of a Knudsen diffusion membrane is given by the square root of the ratio of molar weights of diffusing components. Surface adsorption and diffusion could also notably contribute to the gas permeation in small-pore-diameter membranes. Regarding the wide range of pore diameters or defective membranes, all the contributions mentioned have to be taken into consideration [47,49].

For simplified calculations, the pore flow model based on the Darcy Law is commonly applied, as Equation (3) describes:

(3) $$J=-kC_{i}\frac{d(p)}{d(x)}$$

where *dp*/*dx* is the pressure gradient across the membrane, *c_i_* is the concentration of component *i*, and *k* is a parameter used to describe the nature of the material.

### 4.1. Gas Transport in Dense Membranes

As mentioned previously, the gas permeation of dense polymeric membranes is described by the solution–diffusion model [50]. The gas transport occurs when the gas molecules are dissolved in the membrane surface, and molecules diffuse across the membrane by way of the cavities present in the polymer. Certainly, the permeability results from the contribution of these two mechanisms, and can be described by Equation (4):

(4) P=D⋅S

where *P* is the permeability, *D* is the diffusion coefficient, and *S* is the sorption coefficient. The sorption coefficient is a thermodynamic factor that depends on the gas condensability and the material physicochemical properties. The permeability is also related to the gas partial pressure. In particular, the gas dissolved in a polymer can be considered to be directly proportional to the partial pressure, being expressed by Henry’s Law. The diffusion coefficient is a kinetic factor that is expressed by Fick’s laws [51]. This diffusion coefficient provides the input of the required energy for the gas to accomplish its passing through the polymer and its intrinsic packing. Nevertheless, this coefficient is also determined by the size and the shape of the gas molecules [52].

Moreover, the performance of dense membranes is also evaluated from the ideal selectivity, which is expressed by Equation (5). Typically, the selectivity (*α*) is defined as the relation of the permeability for the components *A* and *B*. According to Equation (4), this can be described using the diffusivity coefficients, known as “diffusion selectivity”, and the ratio between the sorption coefficients, known as “adsorption selectivity” [51].

(5) $$\alpha_{A/B}=\frac{P_{A}}{P_{B}}=\frac{D_{A}}{D_{B}}\cdot \frac{S_{A}}{S_{B}}$$

**Figure 6:**
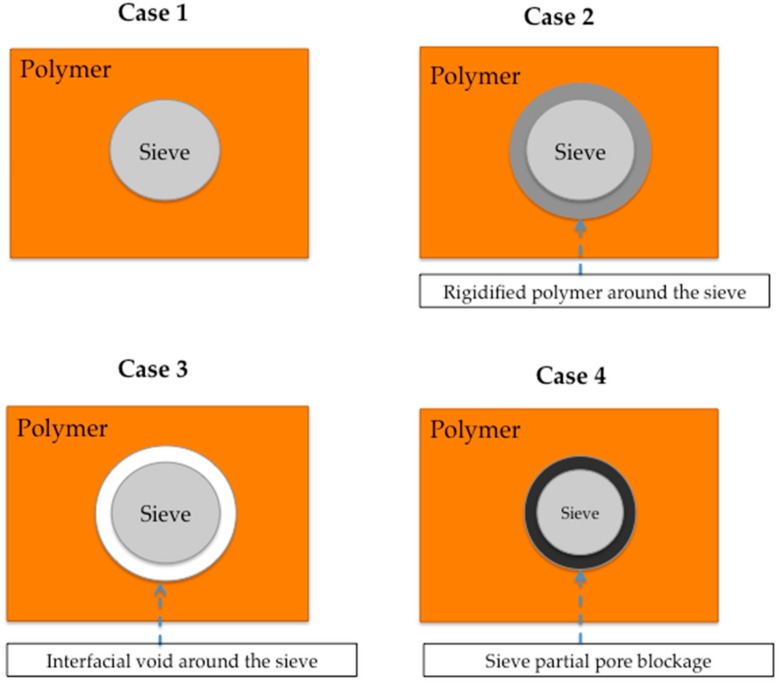
General description of different structures at the polymer–zeolite interface region [53].

However, the aforementioned description of dense pristine polymeric membranes will surely differ when incorporating inorganic materials (e.g., zeolites) into these polymer matrixes. In particular, the zeolites modify the bulk polymer matrix—crucially, the interface region between the polymer and zeolite surfaces—to reach an enhanced performance over the pure polymer. Fundamentally, two factors are highly important to the formation of the interphase: (i) the nature of the polymer–zeolite interaction and (ii) the stress carried out during the MMM preparation. Figure 6 provides a general overview of the different types of structures that can arise at the polymer–zeolite interface region.

For instance, the ideal interphase morphology occurs when a homogeneous blend between the polymer and the zeolites can be obtained (Case 1). The polymer chain rigidification appears when there is shrinkage stress produced in the solvent removal, producing a region in the external polymer phase around the zeolite (Case 2). Poor compatibility between zeolite and polymer, known as “sieve-in-a-cage”, generates the formation of voids at the interfacial region (Case 3). Ultimately, the rigidified polymer chains may partially block the surface pores of the zeolites (Case 4) [53,54]. Certainly, the interaction directly related to the compatibility between the polymer and the zeolite is a key factor of the chemical nature of the polymer and zeolite surface, which can be neutral, attractive, or repulsive [55]. Obviously, these different structures play an important role in the gas separation performance of the membranes. For instance, the increase of rigidity of the polymer (Case 2) matrix results in higher selectivity values, which is accompanied by low gas permeabilities. This is due to the fact that rigidified polymer close to the inorganic material (filler) may have enhanced diffusivity due to the lower polymer chain mobility; this means that the diffusivity gradient between larger and smaller gas molecules may be increased [50]. On the other hand, pore blockage by polymer chains on the surface region of the filler is also critical (Case 4), particularly for zeolites. If pore blockage takes place, the zeolite (NaX) could be extensively excluded from the transport as a result of pore filling by the polymer chains; thereby, a minimal improvement in performance could be reached. In addition, pore blockage generally comprises and is accompanied by chain rigidification. The blockage may reduce a part of the pores of some zeolites (e.g., 5A or beta) to approximately 4 Å, which can discriminate the gas pair of O_2_ and N_2_ [56]. Generally, pore blockage of porous fillers produces a decrease in gas permeation, but the effect on the selectivity is different for each different inorganic material used. According to Chung et al. [50], pore blockage greatly decreases the selectivity when the original filler pore size is comparable to the kinetic diameter of the fast gases tested, e.g., 4A zeolite towards O_2_/N_2_ and CO_2_/CH_4_ gas mixtures. On the contrary, pore blockage can increase the selectivity when the original filler pore size is larger than the kinetic diameter of tested slow gases, e.g., zeolite 5A and beta towards O_2_/N_2_ and CO_2_/CH_4_ gas mixtures.

### 4.2. Strategies to Reach Optimal Interface Morphology

Nowadays, researchers are trying to face the issue of interface voids to obtain membranes with guarantee enhanced performance. As is well known, the suitable selection of a polymer is crucial; for instance, the choosing of a polymer which presents a flexible backbone chain at the membrane preparation temperature should considerably avoid de-wetting [50]. It is important to mention that the de-wetting produces low adhesion between polymer and filler, and thus results in nonselective voids at the interface [14]. The attractive force between the particle and polymer plays an important role; this may be helpful to preparing MMMs with a perfect interface. At this point, Matrimid^®^ displays considerable attractive force towards zeolite 4A [57].

In the case of zeolite-based MMMs, the use of a poly (imide siloxane) copolymer could produce good contact with the zeolite surface [58], obtaining superior-performing MMMs. This is because poly (imide siloxane) copolymer provides flexibility from the flexible siloxane component. Furthermore, the incorporation of a group in the polymer chains reacting with hydroxyl groups, which zeolite surfaces normally have, could be effective in preventing interface void formation during the polymer chain shrinkage. On the other hand, the grafting of coupling agents (commonly amino silane) on zeolite surfaces could also promote good adhesion [14,59]. In particular, silane groups can react with the hydroxyl groups of the zeolite surface, while the amino groups can react with functional groups of the polymers (e.g., imide group in Matrimid^®^), hence possibly forming covalent bonding between the two phases. The attachment of novel agents, such as thionyl chloride, can also favor the enhancement of the filler–polymer interface region; certainly, it has been reported that this agent produces special zeolite surface morphology leading whiskers. This particular roughness provides enhanced interaction at the polymer/particle interface via induced adsorption and interlocking of polymer chains in the whisker structure [60].

Finally, when dealing with the promotion of a good filler–polymer interface, the membrane preparation procedure is also a critical factor. The surface priming protocol has been suggested. The priming implies the coating of the particles with a small but sufficient amount of the polymer (commonly with 5–10 wt % polymer solution). This is immediately followed by a sonication process, which helps to avoid particle agglomeration (i.e., zeolites) and thus facilitates membrane formation [18]; for example, MMMs with coated 4A zeolite have shown enhanced selectivity in O_2_/N_2_ separation compared to the pristine using this priming procedure [50]. This procedure is included as a feasible tool to prepare compelling MMMs; however, there are some other factors that influence mixed matrix membrane fabrication, and which represent a challenge to obtaining the desired morphology, gas separation properties, and (chemical/mechanical) stability [14].

## 5. Zeolites as Filling Material in the Preparation of MMMs Based on Matrimid^®^5218

### 5.1. Beginnings of Incorporating Zeolites into Matrimid^®^

Since time ago, different types of MMMs based on Matrimid^®^ were prepared using zeolites [46,61]; Table 4 depicts a summary of the main zeolites that have been incorporated in this commercial PI, and highlights the remarks from each study.

For instance, Yong et al. [46] proposed the addition of different zeolites (4A, 5A, 13X, NaY) into Matrimid^®^. Particularly, the MMMs with the 13X zeolite considerably increased the permeability of Matrimid^®^ for He, CO_2_, O_2_, and N_2_; on the contrary, the permeation of CH_4_ was restricted, leading to better CO_2_/CH_4_ selectivities than those from the pristine PI membranes.

Jiang et al. [62] used nano-sized beta zeolites for the preparation of mixed matrix single- and dual-layer asymmetric hollow fiber membranes. The MMMs displayed high permeability values but a low separation factor for O_2_/N_2_ (about 0.96). This high permeance of the membranes was attributed to the presence of interface defects that did not display any selectivity. The defects can be explained by (i) poor contact between the filler material and the PI, or (ii) low polymer concentration (20%) in the dope solution.

Lately, Jiang et al. [63] also prepared hybrid hollow fiber membranes by using PSF/beta zeolite/Matrimid^®^, which had a high separation factor (about 77) for the He/N_2_ gas pair. Compared to their previous study [62], these MMMs reached a considerable improvement in the O_2_/N_2_ separation factor (about 6.1) [63]. Moreover, PSF–beta zeolite MMMs as an outer layer and the PI as inner layer [64] displayed selectivity values for O_2_/N_2_ of up to 9.0; such membranes also showed high CO_2_/CH_4_ selectivity (about 128). In order to reduce possible interface defects, it is common to immerse the membranes into organic solvents. Jiang et al. [65] used a p-xylenediamine/methanol soaking method aiming to suppress the polymer–zeolite interface defects. This procedure was evaluated on MMMs that had an inner pure Matrimid^®^5218 layer and outer thin PSF/beta zeolite layer in dual-layer composite hollow fibers. These MMMs (at 30 wt % zeolite loading) presented a 30% and 50% higher selectivity for O_2_/N_2_ and CO_2_/CH_4_, respectively, than did the pure PSF/Matrimid^®^ hollow fiber membranes. These results seem to affirm that the soaking method was able to decrease the interface defects of those membranes.

Zeolite 4A was used to fill a PES/Matrimid^®^ polymer blend by Ismail et al. [66], and the generated membranes were proposed for O_2_/N_2_ separation. At 30 wt % zeolite loading, the membranes provided the best performance with selectivity values of 4.5. Moreover, the addition of mesoporous materials, like ZSM-5, has been demonstrated to enhance the separation performance of Matrimid^®^ [67]. ZSM-5 is synthetized by zeolite seeds, as framework-building units, resulting in a material that possesses mesopores and micropores. In this sense, the material displays synergistic characteristics due to combining the advantages of zeolites (size- and shape-selective adsorption) and mesoporous molecular sieves (improved interfacial properties). In other words, ZSM-5 nanoparticles provide size and shape selectivity. The incorporation improved the ideal selectivity for H_2_/N_2_ separation, from 79.6 for the unfilled membrane to 143.0 using 10 wt % of mesoporous material only, while the O_2_/N_2_ selectivity increased from 6.6 up to 10.4 at 20 wt % of ZSM-5 [67]. Furthermore, the MMMs containing 20 wt % displayed a considerable increase in H_2_/CH_4_ selectivity values, e.g., from 83.0 up to 169.0. It was seen that the mesopores of the ZSM-5 provided a good interface contact between the nanoparticles and polymer. It is quite possible that polymer chains could penetrate into the mesopores, resulting in a good and stable interface [67,68].

### 5.2. Recent Developments on Incorporating Zeolites into Matrimid^®^

More recently, the mesoporous ZSM-5 was again filled into other Matrimid^®^ blend membranes, like polysulfone (PSF)-Matrimid^®^ [61]. The filled membranes showed high permeability values for several gases, such as CH_4_, N_2_, O_2_, and CO_2_, compared with the unfilled membranes. However, their performance did not surpass the CO_2_/CH_4_ and O_2_/N_2_ selectivities reported for the pristine PI. Commonly, the addition of nanoparticles is expected to increase the permeability due to the fact that they increase the free volume fraction of the polymer matrix, but they also tend to produce chain packing disruption, and the porous materials increase the diffusivity of gases. These phenomena have been seen by Peydayesh et al. [72], who added another zeolite-based material into the PI, as silicoaluminophosphate (SAPO)-34. This material has particular shape selectivity as well as molecular sieving properties related to its pore diameter of 0.38 nm, which is near to the CH_4_ kinetic diameter. In addition, it presents a strong CO_2_ adsorption capacity, which makes it attractive for the separation of CO_2_ from natural gas. The MMMs displayed a CO_2_/CH_4_ selectivity around 67—a higher value than that for the pristine Matrimid^®^ membrane (34). The addition of this filler allowed an increase in the permeation of CO_2_, and simultaneously decreased the permeation of CH_4_, thereby enhancing selectivity. It is important to mention that the MMMs were also more thermally stable. In a different study, SAPO-34 also promoted an increase in the permeability values of Matrimid^®^ for H_2_, CO_2_, N_2_, and CH_4_ to about 40.2, 12.5, 1.19, and 1.34 [76]; typically, Matrimid^®^ displays 30.3, 9.54, 0.70, and 0.32 Barrer for these gases, respectively. Unfortunately, the MMMs did not maintain or increase the initial selectivity of Matrimid^®^. This can be attributed to poor interactions between the filler and the PI that lead to the production of interfacial voids. According to the authors, this transient free volume can be attributed to polymer chain mobility [76]; certainly, these interfacial voids may represent a new pathway for the gases to pass through the membrane [77,78], which is in agreement with the nonimprovement of selectivities due to these voids which tend to be poorly selective. Another zeolite-type material (DDR) was also filled into Matrimid^®^ for hydrogen purification applications [75]. This material contributed to enhancing the H_2_ permeability of Matrimid^®^ (17 Barrer) up to 34.9 Barrer in MMMs (using 20 wt % filler loading). Meanwhile, the H_2_/CH_4_ selectivity reached up to 375 (from an initial value of 129), meaning an enhancement of 189% in this property. According to the authors, the improvement was associated with the good contact at the polymer and zeolite interface, and the good molecular sieving effect that this filler displays.

Today, the chemical modification of the filling materials is a current approach seeking better features during their addition into the polymeric matrix. Chen et al. [59] performed the chemically grafted modification of zeolite (AU/EMT intergrowth zeolite) to prepare MMMs with cross-linked Matrimid^®^ (by adding bis(3-aminopropyl)-tetramethyldisiloxane (APTMDS)). Basically, the chemical modification changes the surface density, micropore volume, and CO_2_ adsorption capacity. The addition of the modified zeolite and the cross-linking procedure of the PI resulted in MMMs that displayed selectivity values of 41.4, from a pristine Matrimid^®^ membrane with a CO_2_/CH_4_ selectivity of 28. Indeed, the MMMs showed much better separation performance and thermal stability than did the pure unfilled membranes. Similarly, another mesoporous zeolite-type filler, MCM-41 [79,80], was chemically modified [81]. Practically, the mesoporous spheres were functionalized with sulfonic (–SO_3_H) groups; these functionalized MMMs reached an up to 31% increase in CO_2_ permeability, contributing to a 14% increase in CO_2_/CH_4_ selectivity [81]. In theory, the polar groups (–SO_3_H) tend to increase the CO_2_ solubility in membranes due to interaction with the CO_2_ quadrupole. Ebadi Amooghin et al. [39] prepared MMMs by incorporating micro- and nano-porous sodium zeolite-Y (NaY zeolite). Matrimid^®^ membranes with enhanced CO_2_ transport were obtained by embedding this filler [39]. Particularly, this NaY zeolite was chosen because of its larger pore size compared to the other types of zeolites, which can facilitate the activated diffusion of gas molecules. Additionally, it gives superior adsorptive molecular transport by differences in the adsorptivities of the gases. These membranes demonstrated an outstanding performance for CO_2_/CH_4_ separations: the CO_2_ permeability was increased more than twofold while the separation factor was enhanced by 20%, from 36.3 in Matrimid^®^ to 43.3 in MMMs. Meanwhile, Loloei et al. [73] considered again the incorporation of ZSM-5 into Matrimid^®^ but a previous blending was carried out. The PI was blended with polyethylene glycol (PEG). The ternary MMMs revealed that the CO_2_ permeability and CO_2_/CH_4_ selectivity of pure Matrimid^®^ were significantly enhanced. Specifically, the CO_2_ permeability of the ternary MMMs (Matrimid^®^/PEG (95:5) with 5 wt % ZSM-5) was increased about 50% (from 7.6 to 11.5 Barrer) and CO_2_/CH_4_ selectivity about 72% (from 34.9 to 60.1). It is clear that the enhancement of both properties is fully supported. The novelty of the synthesis of hybrid ternary membranes can be a promising approach to developing new membranes with better performances considering no modification of the filling material, but correctly choosing an additive, like PEG, which tends to offer some CO_2_ affinity, favoring its permeation.

**Figure 7:**
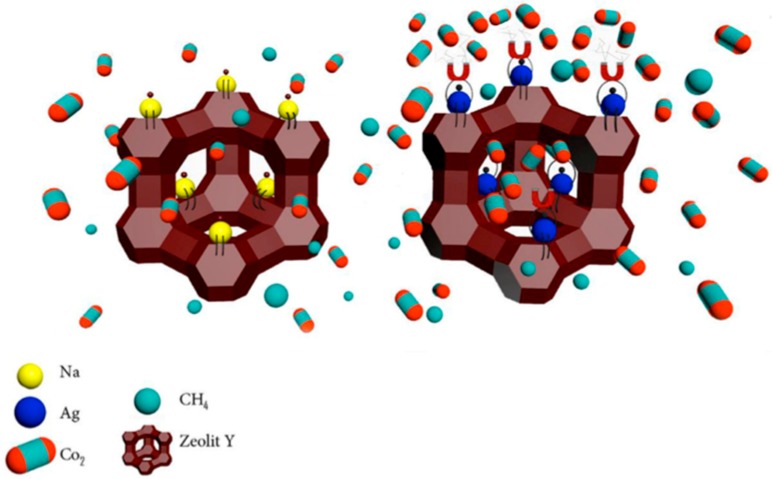
Schematic drawing of the CO_2_-facilitated transport through the modified zeolite-Y [82].

Zeolite-Y was ion-exchanged by introducing silver (Ag) cations into the framework of micro-sized nano-porous sodium zeolite-Y using a liquid-phase ion exchange method [82]. This novel filler combined the effect of the facilitated transport mechanism of Ag^+^ ions as well as the intrinsic surface diffusion mechanism of the Y-type zeolite. Figure 7 represents the CO_2_-facilitated transport via Ag^+^ ions located at the external and internal surfaces of zeolite Y.

The incorporation of Ag^+^ ion-exchanged zeolite-Y increased CO_2_ permeability values (about 123%, from 8.34 for pure Matrimid^®^ to 18.62 Barrer for Matrimid^®^/AgY) and CO_2_/CH_4_ selectivity (about 66%, from 36.3 for Matrimid^®^ to 60.1 for Matrimid^®^/AgY) [82]. Similarly, Mundstock et al. [83] exchanged the Na^+^ of the as-synthesized NaX zeolite particles for metal ions with higher ionic potentials, such as Co^2+^; the resulting MMMs presented enhanced mixed gas separation factors for H_2_/CO_2_ separation, e.g., from 4.0 to 5.6 for NaX/Matrimid^®^ and CoX/Matrimid^®^, respectively. On the other hand, Gong et al. [84] developed mixed matrix membranes which contained inorganically surface-modified 5A zeolite. First, 5A zeolites were successfully prepared via a facile treatment in an aqueous phase through which nanostructures of Mg(OH)_2_ were grown on the zeolite surfaces. These zeolites had enhanced surface roughness. Indeed, the modification of the zeolite enhanced the zeolite–polymer adhesion, together with the CO_2_/CH_4_ separation performance. As was seen, a strong increase in CO_2_ permeability (about 120%), from 10.2 up to 22.4 Barrer, was observed in Matrimid^®^+20 wt % 5A membrane, which also displayed a slightly enhanced CO_2_/CH_4_ selectivity (36.4 from 33.6 in Matrimid^®^). In contrast, the nonmodified 5A decreased the CO_2_/CH_4_ selectivity of Matrimid^®^ membrane due to defects formed at the zeolite/polymer interfaces. The most recent study found relating the use of zeolites in Matrimid^®^ matrix for the preparation of MMMs has been presented by Ebadi Amooghin [85]. They proposed a new synthesis strategy for preparing novel hybrid host–guest nanocomposites by encapsulating a metal–organic complex of a transient metal such as cobalt (Co) in zeolite Y cavities with the ship-in-a-bottle synthesis method. Once synthesized, the nanoparticles were incorporated into the Matrimid^®^ aiming for CO_2_/CH_4_ separation. According to the results, these MMMs provided a greater separation performance, e.g., MMMs containing 15 wt % filler loading had a CO_2_ permeability of about 17 Barrer and CO_2_/CH_4_ selectivity of about 102, which were more than two- and three-fold those of pure Matrimid^®^, which had a permeability about 6.6 Barrer and CO_2_/CH_4_ selectivity of 30 [85]. This enhancement is attributed to the presence of Co^2+^ due to the fact that it presents better intermolecular interaction between the CO_2_ and the complex molecule. Moreover, the authors also confirmed excellent interactions between nanoparticles and the PI, displaying enhanced interfacial adhesion. Indeed, the good PI–filler compatibility can be attributed to the weak acid–base Lewis interactions between the PI carbonyl groups and the Co functionalized groups on the zeolite surface [85]. In this sense, it is quite possible that these MMMs can be good candidates to go forward for other types of separations.

**Figure 8:**
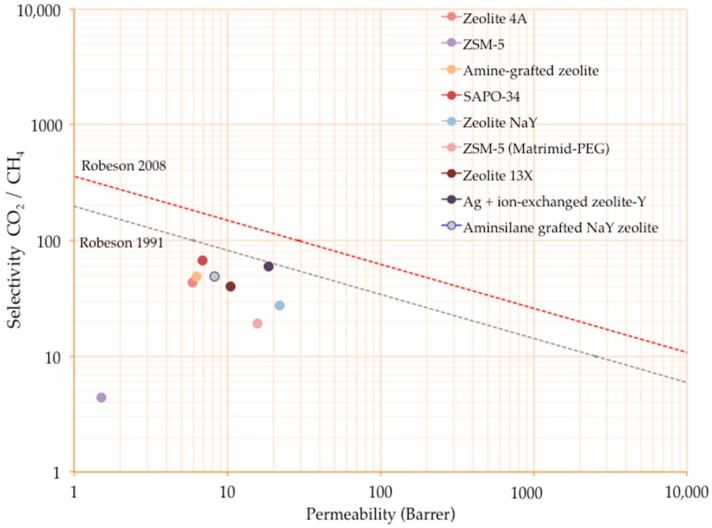
Status of MMMs based on Matrimid^®^ and zeolites on Robeson plot 1991–2008.

To date, most of the MMMs based on Matrimid^®^ containing zeolite-based fillers have displayed compelling gas separation performance. When relating their location on the Robeson trade-off, zeolite 4A, amine-grafted zeolite, SAPO-34, zeolite NaY, ZSM-5 (also containing an additive like PEG in the polymer matrix), zeolite 13X, and some chemically modified zeolites (e.g., Ag^+^ ion-exchanged zeolite-Y and aminosilane-grafted zeolite) have demonstrated significant improvements over the performance of Matrimid^®^, allowing a performance located on the border of the Robeson plot established in 1991, but still far from the one revisited in 2008 [9], as Figure 8 describes. It is important to note that such relationships have been proposed from data of testing pristine polymeric membranes [9].

The success of these MMMs in nearly overcoming this relationship is clearly attributed to the type of zeolites used—and their adsorption capacity—as an adsorbent. The adsorption is defined as the uptake of a component (the so-called adsorbate, e.g., gas) in the gas–solid interface onto the adsorbent. At this point, CO_2_ capture using absorbents (in solid state) is recognized as one of the most promising approaches for its recovery and separation [86]. This is because adsorption can diminish the energy requirements and cost of its separation. As is well documented, zeolites are considered as a good CO_2_ adsorbent together with other filling materials (e.g., activated carbon and alkali metal-based materials) [87]. For instance, specific zeolites such as NaX, 5A, and 13X possess CO_2_ adsorption capacities of about 263 [88], 222 [89], and 324 mg CO_2_/g adsorbent [90], respectively. Definitely, the CO_2_ uptake of the zeolites depends crucially on the Si/Al ratio [91]. At this point, it is quite possible that coming studies will be focused on finding a suitable Si/Al ratio of the zeolites that could provide higher CO_2_ adsorption. In this way, zeolites can guarantee a better effect towards CO_2_ transport, influencing the separation efficiency.

Finally, as reported previously [4], Matrimid^®^-based MMMs using zeolites have also demonstrated their ability to separate other gas mixtures e.g., H_2_/CH_4_, CO_2_/N_2_, or H_2_/N_2_ (see Table 4). Most of these MMMs, containing zeolite 4A, ZSM-5, zeolite 13X, and MCM-41, are also quite close to surpassing the Robeson limit (1991).

## 6. Future Trends and Concluding Remarks

This review compiles the initial and recent developments in the use of zeolites and zeolite-based materials incorporated into Matrimid^®^ membranes for gas separation. All these literature inputs have demonstrated the potentialities of zeolites regarding the enhancement of Matrimid^®^. To date, Matrimid^®^–zeolite MMMs have been mainly considered for CO_2_/CH_4_ separations; however, they have displayed acceptable performances for other gas separations applications, such as O_2_/N_2_, He/N_2_, H_2_/N_2_, H_2_/CH_4_, and H_2_/CO_2_, giving an outlook of the versatility of such membranes. At this point, it is crucial to adopt a proper strategy in exploiting the synergistic beneficial features of the advanced materials, processes, and modification techniques in order to achieve Matrimid^®^ membranes with desirable performance. When attempting to enhance some other properties of Matrimid^®^, e.g., thermal stability, the zeolites can contribute as well.

It is likely that the chemical modification of zeolites, as a promising tool for enhancing the features of the existing ones, will be explored in coming years according to the resulting great separation performances. Moreover, chemical modification and the synthesis of new zeolites are promising alternatives in order to prepare MMMs which can display better compatibility or interactions (e.g., interfacial adhesion) between this PI and zeolites, contributing to suppressing the interfacial voids. However, some other procedures, like the p-xylenediamine/methanol soaking method, priming, and applying high processing temperatures close to T_g_ (polymer) to maintain the polymer chain flexibility, can be useful for this purpose, too [53].

It is clear that the researchers' interest is currently focused on MMMs, which imply multiple components' role in the interfacial area. However, the formation of these MMMs and their mechanism need further intensive understanding. Moreover, the next generation of MMMs could address the use of nano-sized materials avoiding the formation of clusters (agglomeration). This can surely guarantee the exploitation of their separation properties. In addition, the incorporation of nano-fillers could allow the preparation of thinner membrane layers and better filler distribution. As is well documented, smaller materials provide more polymer–particle interfacial area, and thus improve the polymer–particle interface contact. Furthermore, the shape and morphology also play an important role. 

To date, gas separation tests for MMMs have been mainly performed in single or binary mixtures, which is a suitable starting point; however, practical applications (e.g., natural gas purification) always imply complex gas mixtures that contain multiple components (e.g., acids, water, and inert gases). This will surely change the expected separation performance of those MMMs. It is time to encourage the testing of membranes using complex gas mixtures for a better approximation. Particularly, the filler stability at these real conditions has to be evaluated [92]. Finally, such an evaluation will provide clear input about the potentialities of Matrimid^®^–zeolite MMMs for industrial use, which has not occurred until now. Nevertheless, there is still much missing research and development for MMMs based on zeolite–Matrimid^®^ mixed matrix membranes for gas separation.

## Figures and Tables

**Table 1:** Features of some filler materials used for MMM preparation [14,15].

Zeolites	MOFs	Silicas	Carbon Molecular Sieves
Fixed pore size	Cations interconnected by organic anions	Directly alter the molecular packing of the polymer chains	High adsorptivity capacity
High temperature stability	Rather flexible and dynamic frameworks	Increase the free volume of polymers	Relatively wide opening with constricted apertures
High stability in humidity	Coordinative bonds	Nonpermeability of the nonporous silica particles	Better affinity to glassy polymers
Limitations for modification	Flexible pore size, soft structure	Probable weak interaction silica–polymer	Good adhesion at interfaces
Pore size crystallographically controlled	Not well-defined molecular sieving	High possibility to produce interfacial voids	High productivity with excellent separation
Great potential as supported thin film	Low temperature stability	Possibilities for surface modification (e.g., silane coupling)	Well-defined molecular sieving
Not thermodynamically most stable but dense structures	Poor stability in humidity	-	Great potential for MMMs
Well-defined molecular sieving	Thermodynamically unstable	-	-
Good sorption and diffusion properties	A variety of possibilities for modification	-	-
-	Offer accessible open metals	-	-
-	Great potential for MMMs	-	-

**Table 2:** Features of Matrimid^®^5218 membranes compared to other polymeric membranes for different gas separations. Adapted from [4].

Polymer	T_g_ (°C)	Permeability (Barrer)					Selectivity						FV	*ρ* (g/cm^3^)
		O_2_	H_2_	N_2_	CO_2_	CH_4_	O_2_/N_2_	CO_2_/N_2_	CO_2_/CH_4_	H_2_/CO_2_	H_2_/N_2_	H_2_/CH_4_		
Matrimid^®^	302–310	2.1	27.16	0.28	7.68	0.22	6.4	30	34.91	3.88	97	83.33	0.17	1.2
Polymers of intrinsic microporosity (PIM-1)	399–415	370	1300	92	2300	125	4.0	25	18	0.57	14	10	0.24	0.94
Polysulfone (PSF)	185	1.2	16.4 *	0.20	4.9	0.21	6.0	22.4	23.3	1.53	20	34.4	0.13	1.19
Poly[1-(trimethylsilyl)-1-propyne] (PTMSP)	>250	7200	4200	6890	37,000	18,400	1.7	10.7	4.46	0.53	2.5	0.995	0.34	0.83
Polybenzimidazole (PBI)	435	0.009	0.6	0.0048	0.16	0.0018	2.0	3.5	88.88	3.75	125	333.3	0.11	1.311

* In terms of permeance (GPU units); FV: Free volume; T_g_: Glass transition temperature; *ρ:* density.

**Table 3:** Features of the main gas molecules used for gas separation. Adapted from [5].

Molecule	Kinetic Diameter (Å)	Polarizability (Å^3^)	Dipole Moment (D)	Quadrupole Moment (D Å)
CO_2_	3.30	2.650	0.000	4.30
CH_4_	3.76	2.600	0.000	0.02
H_2_	2.89	0.80	0.000	0.66
O_2_	3.47	1.600	0.000	0.39
CO	3.69	1.95	0.112	2.50
N_2_	3.64	1.760	0.000	1.52

**Table 4:** Zeolite materials incorporated into Matrimid^®^ for gas separation applications.

Type of Zeolite	Filler Loading	Evaluated Application	Conditions	Performance	Remark of the Study	Reference
Zeolite 4A	15 wt %	Separation CO_2_/CH_4_	Single gas permeation, 10 bar, 30 °C.	CO_2_: 5.9 Barrer CH_4_: 0.1 Barrer CO_2_/CH_4_: 43	Good interaction between zeolite and polymer, enhancing the separation performance.	[69]
ZSM-5	10 wt %	Separation CO_2_/CH_4_, O_2_/N_2_	Single gas permeation, conditions: 2–5 bar, 35 °C.	N_2_: 0.2 Barrer O_2_: 0.7 Barrer CH_4_: 0.3 Barrer CO_2_: 1.5 Barrer CO_2_/CH_4_: 4.4 O_2_/N_2_: 3.0	The MMMs displayed higher permeability than the pristine polymer.	[61]
Zeolite 4A	30 wt %	Separation CO_2_/N_2_, He/N_2_, H_2_/He, H_2_/CO_2_	Single gas permeation, conditions: 10 bar, 25 °C.	H_2_: 83 Barrer CO_2_: 262 Barrer N_2_: 140 Barrer He: 38 Barrer CO_2_/N_2_: 50.6	Enhanced permeability for He, H_2_, CO_2_, and N_2_ increasing with zeolite loading.	[70]
ZSM-5	20 wt %	Separation CO_2_/N_2_	Single gas permeation, conditions: 10 bar, 25 °C.	H_2_: 147 Barrer CO_2_: 423 Barrer N_2_: 180 Barrer He: 108 Barrer CO_2_/N_2_: 86.2	Enhanced permeability for He, H_2_, CO_2_, and N_2_ increasing with zeolite loading.	[70]
Zeolite 13X	30 wt %	Separation CO_2_/N_2_	Single gas permeation, conditions: 10 bar, 25 °C.	H_2_: 178 Barrer CO_2_: 378 Barrer N_2_: 185 Barrer He: 111 Barrer	Enhanced permeability for He, H_2_, CO_2_, and N_2_ increasing with zeolite loading.	[70]
Amine-grafted zeolite	25 wt %	Separation CO_2_/CH_4_	Single gas permeation, conditions: 150 psi, 35 °C.	CO_2_: 6.3 Barrer CH_4_: 0.1 Barrer CO_2_/CH_4_: 48.5	Cross-linked Matrimid^®^ and modified zeolite displayed a considerable enhancement towards CO_2_/CH_4_ separation.	[59]
Zeolite 4A	30 wt %	Separation CO_2_/N_2_, O_2_/N_2_, H_2_/N_2_	Single gas permeation, conditions: 8 bar, 30 °C	H_2_: 101.6 Barrer CO_2_: 48.3 Barrer O_2_: 11.1 Barrer N_2_: 2.0 Barrer CO_2_/N_2_: 23.3 O_2_/N_2_: 5.3 H_2_/N_2_: 49.1	The MMMs showed enhanced permeability for all gases.	[71]
SAPO-34	20 wt %	Separation CO_2_/CH_4_	Single gas permeation, conditions: 10 bar, 25 °C	CO_2_: 6.9 Barrer CH_4_: 0.1 Barrer CO_2_/CH_4_: 67	The MMMs displayed enhancements for both permeability and selectivity.	[72]
Zeolite NaY	20 wt %	Separation CO_2_/CH_4_	Single gas permeation, conditions: 2 bar, 35 °C.	CO_2_: 22 Barrer CH_4_: 0.8 Barrer CO_2_/CH_4_: 27.6	The CO_2_ permeability was enhanced more than twofold by incorporating the zeolite	[39]
ZSM-5	5 wt %	Separation CO_2_/CH_4_	Single gas permeation, conditions: 10 bar, 35 °C.	CO_2_: 15.7 Barrer CH_4_: 0.8 Barrer CO_2_/CH_4_: 19.2	The MMMs displayed enhancements for both permeability and selectivity.	[73]
Zeolite 13X	30 wt %	Separation CO_2_/CH_4_	Single gas permeation, conditions: 12 bar, 25 °C.	CO_2_: 10.5 Barrer CH_4_: 0.2 Barrer CO_2_/CH_4_: 39.8	The MMMs displayed enhanced separation performance over the pristine polymer.	[74]
Deca-dodecasil 3R (DDR)	20 wt %	Separation H_2_/CH_4_	Single gas permeation, conditions: 10 bar, 35 °C.	H_2_: 34.9 Barrer H_2_/CH_4_: 375.2	The incorporation of the zeolite-type filler enhanced the hydrogen permeability more than 100%.	[75]
